# Biomedical Signal Processing and Health Monitoring Based on Sensors

**DOI:** 10.3390/s25030641

**Published:** 2025-01-22

**Authors:** Sang Ho Choi, Heenam Yoon, Hyun Jae Baek, Xi Long

**Affiliations:** 1 Department of Computer and Information Engineering, Kwangwoon University, 20 Gwangun-ro, Seoul 01897, Republic of Korea; 2 Department of Human-Centered Artificial Intelligence, Sangmyung University, 20 Hongjimun 2-gil, Seoul 03016, Republic of Korea; h-yoon@smu.ac.kr; 3 Department of Biomedical Engineering, Soonchunhyang University, Asan-si 31538, Republic of Korea; hjbaek@sch.ac.kr; 4 Biomedical Diagnostics Lab, Department of Electrical Engineering, Eindhoven University of Technology, 5612 AZ Eindhoven, The Netherlands; x.long@tue.nl

The healthcare industry is undergoing rapid transformation driven by advancements in Internet of Things (IoT) technologies, particularly in biomedical signal processing and health monitoring. The global COVID-19 pandemic, coupled with an aging population and the rising incidence of chronic diseases, has significantly accelerated the development of IoT-based health monitoring services. Consequently, the healthcare paradigm is shifting from conventional diagnosis and treatment toward prevention, prediction, and personalization. This Special Issue, **“Biomedical Signal Processing and Health Monitoring Based on Sensors”**, introduces innovative studies on these changes. The contributions in this Special Issue are characterized by a focus on practical medical tasks, real-time data analysis, and predictive capabilities. The 15 featured papers present methods for health monitoring and disease prevention using a variety of sensors and data types, signal processing methodologies, and artificial intelligence (AI) models.

This Special Issue includes seven innovative papers on neural signal processing related to electroencephalography (EEG)-based brain activity analysis for diagnostics, emotion recognition, and brain–computer interfaces. Khare et al. [1] present an intelligent motor imagery detection system based on EEG signals, employing robust tunable Q wavelet transform with evolutionary optimization algorithms for adaptive parameter tuning. The approach achieves superior accuracy (99.78%) using a least-squares support vector machine classifier, outperforming existing methods on the same dataset. Zhang et al. [2] propose a novel method for emotion recognition based on EEG, integrating long short-term memory with feature pyramid networks. The method achieves high classification accuracies for valence (90.05%) and arousal (90.84%) by incorporating spatial topology information with adaptive channel weighting, outperforming the performance of previous approaches. Khaleghi et al. [3] introduce a convolutional neural network-based generative adversarial network (CNN-GAN) framework designed to extract salient arithmetic data from EEG signals, particularly during visual stimulation using MNIST digit categories. The method achieves a classification accuracy of 95.4% and saliency metrics (Structural Similarity: 92.9%; Pearson’s correlation coefficient: 97.28%), demonstrating its effectiveness in reconstructing visually evoked salient images from neural activity.

Rahmani et al. [4] propose an EEG-based automatic lie detection model that combines type-2 fuzzy sets and deep graph convolutional networks (GCNs). The proposed system achieves over 95% accuracy in distinguishing truths from lies, even in noisy environments, and is noted for its practical applicability in various security and behavioral studies. Peivandi et al. [5] present a deep learning approach using physiological signals such as EEG, electrocardiography (ECG), and electromyography (EMG) to detect multi-level driver fatigue with high accuracy, achieving 89% in a five-level classification scenario. By employing novel architecture integrating type-2 fuzzy sets and GANs, the study offers a robust framework for real-time fatigue monitoring and accident prevention. Jiang et al. [6] investigate the efficacy of scent interventions, such as peppermint, grapefruit, and lavender, in mitigating driver fatigue using EEG metrics and subjective fatigue assessments. The study demonstrates significant reductions in fatigue levels with these scents, highlighting grapefruit for its immediate relief and lavender for its prolonged effects, providing insights into odor-based solutions for road safety enhancement. Ardabili et al. [7] propose an innovative approach for automatically detecting driver fatigue based on EEG signals, integrating GANs with GCNs. Their method achieves high accuracy in multi-class fatigue classification and incorporates a robust data collection methodology using a driving simulator, offering significant advancements in real-time fatigue monitoring technology.

In addition, this Special Issue includes eight innovative studies employing advanced sensor technologies and AI frameworks to address diverse health monitoring and intervention challenges. The contributions encompass a broad range of applications, including vital sign analysis, mental health and stress assessment, ergonomic optimization, sleep enhancement, and rehabilitation, demonstrating their potential to advance diagnostic precision and personalized healthcare solutions. Choi et al. [8] introduce a deep learning framework based on CNNs for real-time vital sign monitoring, focusing on respiration and heart rates through non-contact impulse radio ultra-wideband radar. They achieve robust performance by integrating one-dimensional and two-dimensional signal analysis, offering potential applications in long-term vital sign monitoring during sleep. Lee et al. [9] propose a method for reducing the measurement time required for photoplethysmography-based stress index calculation using machine learning models like Extra Tree Regressor and Gradient Boost Regressor. The study achieves a significant reduction in measurement time (from 60 s to 10 s) while maintaining high accuracy, enabling practical use in wearable health monitoring. Zhang et al. [10] propose AVTF-TBN (Audio, Video, and Text Fusion-Three Branch Network), an innovative framework that integrates multimodal data for detecting depression risk. The proposed framework achieves the best performance across multiple metrics, including F1 score, precision, and recall, thereby highlighting the efficacy of sensor-based data and a comprehensive emotion elicitation paradigm for mental health assessments.

Oliosi et al. [11] investigate the differences in real-time variability and complexity of sitting behaviors between office workers experiencing chronic spinal pain (CSP) and those without pain. Several sensors are used in this study, including smartphone inertial sensors (accelerometer, gyroscope, and rotation vector), smartwatch heart rate sensors, and surface EMG sensors. The study finds that CSP participants exhibit less complex and more predictable trunk movements, emphasizing the potential of ergonomic and adaptive interventions to address spinal health risks. Radomski et al. [12] assess a novel magnetic induction sensor that facilitates unobtrusive respiration monitoring within cars, enabling the measurement of drivers’ breathing rates through the seat backrest. The study demonstrates the sensor’s high accuracy (84.45% coverage) in diverse driving conditions, highlighting its potential for detecting fatigue and stress while addressing limitations like motion artifacts and noise in dynamic environments. Yoon et al. [13] introduce a closed-loop auditory stimulation (CLAS) method that synchronizes with individual’ respiratory rhythms to enhance sleep initiation. CLAS demonstrates a reduction in time spent in sleep initiation and shows significant modulation of autonomic nervous system activity, enhancing parasympathetic activity and stabilizing respiratory rhythms for better sleep quality. Furtado et al. [14] examine how gestational age influences pelvic floor muscle activity, plantar contact, and functional mobility in pregnant women at high risk. Their findings reveal a significant decline in pelvic floor muscle myoelectric activity as gestation progressed, while measurements of plantar contact showed no significant changes. These results emphasize the importance of pelvic floor rehabilitation and adaptive interventions to improve mobility and safety for high-risk pregnancies. Dominguez-Vega et al. [15] introduce a classification approach using inertial measurement sensors to distinguish between early-onset ataxia, developmental coordination disorder, and typically developing children. They report improved classification accuracy compared to traditional clinical assessments, with the most critical features being variability in gait movements and hip flexion–extension, which enhance the model’s clinical explainability.

In conclusion, the studies in this Special Issue collectively represent significant advancements in biomedical signal processing and health monitoring based on sensors. The featured studies cover diverse applications, including neural signal analysis, driver fatigue detection, mental health assessment, and non-contact health monitoring, demonstrating the integration of advanced AI models and sensor technologies to achieve high accuracy and real-time applicability. These contributions are expected to pave the way for innovative advances in diagnostics, monitoring, and personalized healthcare solutions, providing valuable insights and practical methodologies to address current challenges.
